# Radio-Histological Correlation of Lung Features in Severe COVID-19 Through CT-Scan and Lung Ultrasound Evaluation

**DOI:** 10.3389/fmed.2022.820661

**Published:** 2022-04-19

**Authors:** Pere Trias-Sabrià, Eduard Dorca Duch, Maria Molina-Molina, Samantha Aso, Marta Díez-Ferrer, Alfredo Marín Muñiz, Jaume Bordas-Martínez, Joan Sabater, Patricio Luburich, Belén del Rio, Xavier Solanich, Jordi Dorca, Salud Santos, Guillermo Suárez-Cuartin

**Affiliations:** ^1^Respiratory Department, Hospital Universitari de Bellvitge, Institut d'Investigació Biomèdica de Bellvitge (IDIBELL), L'Hospitalet de Llobregat, Spain; ^2^Universitat de Barcelona-Campus Bellvitge, L'Hospitalet de Llobregat, Spain; ^3^Pathology Department, Hospital Universitari de Bellvitge, Institut d'Investigació Biomèdica de Bellvitge (IDIBELL), L'Hospitalet de Llobregat, Spain; ^4^Critical Care Department, Hospital Universitari de Bellvitge, Institut d'Investigació Biomèdica de Bellvitge (IDIBELL), L'Hospitalet de Llobregat, Spain; ^5^Radiology Department, Hospital Universitari de Bellvitge, Institut d'Investigació Biomèdica de Bellvitge (IDIBELL), L'Hospitalet de Llobregat, Spain

**Keywords:** COVID-19, lung ultrasonography (LU), biopsy, pathology, radiology

## Abstract

**Background:**

Patients with coronavirus disease 2019 (COVID-19) can develop severe bilateral pneumonia leading to respiratory failure. Lung histological samples were scarce due to the high risk of contamination during autopsies. We aimed to correlate histological COVID-19 features with radiological findings through lung ultrasound (LU)-guided postmortem core needle biopsies (CNBs) and computerized tomography (CT) scans.

**Methodology:**

We performed an observational prospective study, including 30 consecutive patients with severe COVID-19. The thorax was divided into 12 explorations regions to correlate LU and CT-scan features. Histological findings were also related to radiological features through CNBs.

**Results:**

Mean age was 62.56 ± 13.27 years old, with 96.7% male patients. Postmortem LU-guided CNBs were performed in 13 patients. Thirty patients were evaluated with both thoracic LU and chest CT scan, representing a total of 279 thoracic regions explored. The most frequent LU finding was B2-lines (49.1%). The most CT-scan finding was ground-glass opacity (GGO, 29%). Pathological CT-scan findings were commonly observed when B2-lines or C-lines were identified through LU (positive predictive value, PPV, 87.1%). Twenty-five postmortem echo-guided histological samples were obtained from 12 patients. Histological samples showed diffuse alveolar damage (DAD) (75%) and chronic interstitial inflammation (25%). The observed DAD was heterogeneous, showing multiple evolving patterns of damage, including exudative (33.3%), fibrotic (33.3%), and organizing (8.3%) phases. In those patients with acute or exudative pattern, two lesions were distinguished: classic hyaline membrane; fibrin “plug” in alveolar space (acute fibrinous organizing pneumonia, AFOP). C-profile was described in 33.3% and presented histological signs of DAD and lung fibrosis. The predominant findings were collagen deposition (50%) and AFOP (50%). B2-lines were identified in 66.7%; the presence of hyaline membrane was the predominant finding (37.5%), then organizing pneumonia (12.5%) and fibrosis (37.5%). No A-lines or B1-lines were observed in these patients.

**Conclusion:**

LU B2-lines and C-profile are predominantly identified in patients with severe COVID-19 with respiratory worsening, which correspond to different CT patterns and histological findings of DAD and lung fibrosis.

## Introduction

Coronavirus disease 2019 (COVID-19) is a viral infection by novel severe acute respiratory syndrome coronavirus 2 (SARS-CoV-2). Patients with COVID-19 develop interstitial pneumonia, which can lead to respiratory failure (54%) and acute respiratory distress syndrome (ARDS) (31%) (1). The COVID-19 has a global spread all around the globe (2). Monitoring and therapeutic protocols are being modified regarding growing research evidence.

Chest imaging is a valuable tool for diagnosis, clinical management, and guiding therapy. Chest radiography is initially performed for identifying pneumonia and computerized tomography (CT) is recommended for better evaluating ARDS and other potential complications (3). The main radiological findings are multi-lobar asymmetric ground-glass opacity (GGO) and bilateral peripheral consolidations (4–6). When ARDS develops, other findings might be present, such as organizing pneumonia and traction bronchiectasis (7–9). Although chest CT gives relevant information for therapeutic approach, some challenges have been found during the COVID-19 pandemic, including the risk of infection for healthcare workers, the need of space and surface cleaning and disinfection, the life-threatening for critical patients of their transfer, and the inconvenience for monitoring disease progression or worsening in respiratory critical conditions that make difficult the mobilization of patient (3). Lung ultrasound (LU) has been developed as an important tool in monitoring patients with COVID-19, both in intermediate respiratory care units (IMCUs) and in intensive care units (ICUs) (10, 11). LU is easy to use, repeatable, and reproducible, and allows an immediate bedside application which drives to rapid decision-making (12). LU has the ability to identify and monitor an alveolar–interstitial pattern, defined by the presence of convalescents B-lines, white lung, or subpleural consolidations (13–15). Some initial correlations between LU and CT patterns have been described (3). However, the pathological findings behind this radiological imaging of COVID-19-induced lung damage remain elusive.

Histopathological understanding of the disease would be relevant for better targeting therapeutic options. However, performing lung biopsy in severe COVID-19 infection may associate clinical deterioration and potential life-threatening complications. Most pathological findings from autopsies or postmortem biopsies describe diffuse alveolar damage (DAD) with cellular interstitial pattern and fibromyxoid interstitial deposition as well as microthrombi in small vessels (16–19). The different histomorphological patterns of damage could correspond to specific ultrasound and/or tomographic patterns. Characterizing the lung images that correspond to the different histological findings could help in prognostication and optimizing treatments. We aimed to correlate histological COVID-19 features with LU findings and CT-scan features by performing LU-guided transthoracic immediately postmortem core needle biopsy (CNB). Furthermore, we looked for the CT radiological features that were present in the different LU patterns using both as complementary radiological tools.

## Materials and Methods

### Data Collection and Design

This observational prospective study included 30 consecutive patients with severe COVID-19 with progressive worsening despite supportive care who were evaluated through CT scan and LU. All of them were hospitalized in the IMCU or ICU of the University Hospital of Bellvitge, Barcelona, Spain, in April 2020. LU-guided CNB immediately postmortem was performed in 13 patients.

The study protocol was approved by the local ethics committee (N° PR122/20). All procedures performed in this study involving human participants were in accordance with the ethical standards of the institution, and with the 1964 Helsinki Declaration. The inclusion criteria were confirmed COVID-19 diagnosis with a positive polymerase chain reaction (PCR) for SARS-CoV-2, having informed consent by the family for transthoracic biopsy in case of death. The exclusion criteria included a clinical worsening due to other reasons different than COVID-19 lung injury and/or extrapulmonary complications. Demographic, clinical, radiological, and laboratory data were collected, including previous medical conditions, time from the onset of symptoms, blood test analysis, and treatment received. LU findings were described following the international evidence-based recommendations (13).

### LU Exploration

A targeted LU examination was performed by a trained pulmonologist. A Philips Lumify C5-2 transducer was used, equipped with a portable Samsung tablet and the Lumify APP. LU examination was performed with the convex transducer set into lung configuration. During the examination, the portable tablet device and the transducer were wrapped in single-use plastic protection to reduce contamination and improve sterilization. Wireless transducers may be indicated for assessing LU in highly infectious diseases as COVID-19 (3, 13). At the end of the exploration, the tablet and the transducer were sterilized following recent recommendations (20). Ultrasound findings were assessed using the LU score (LUS) as described in the previous studies (11, 13). Briefly, LUS evaluates lung parenchyma following a twelve-zone examination of the thorax (11, 14, 21). Anterior (midclavicular line), lateral (midaxillary line), and posterior (paravertebral line) chest wall regions were divided in two (superior and inferior). Posterior exploration was performed when subjects were able to sit or tilt sideways. When posterior access was not possible, it was substituted with posterior axillary line exploration. The transducer was placed longitudinally to the ribs to enhance pleural and lung exploration. Each region was then scored following recommendations for point-of-care LU (14, 22–24): lung sliding with A-lines or fewer than three isolated B-lines scored 0; multiple well-defined B-lines (B1) scored 1; multiple coalescent B-lines (B2) or white lung scored 2; and subpleural consolidation scored 3. The sum of the scores defined the LUS, ranging from 0 to 36 points (14, 24). Data were directly recorded and saved in video format.

### Computerized Tomography of the Chest

In 30 patients, both LU and CT scan (Aquilion ONE / GENESIS, Toshiba, Barcelona, Spain) were done at the moment of patient worsening. From them, two trained thoracic radiologists described lung parenchyma in all the 12 regions used in LUS. Both radiologists were blinded to LUS and to histological information. CT scans were individually analyzed and then agreed. Endovenous contrast was used in 37%. A total of 279 exploration regions were assessed. GGO, consolidation, organizing pneumonia, septal thickening, and fibrosis were identified. Radiological findings were matched with the LU findings.

### Postmortem CNB of Lung

In those cases that finally died (13 patients), after exploring both hemithoraxes through ultrasound, immediately postmortem CNB guided by LU was performed. All of them were taken from third anterior intercostal space at midclavicular line. A 16G semiautomatic biopsy needle for soft tissue biopsy was used. Between 4 and 6 biopsies were collected in the same intercostal space from each lung.

### Histological Processing of CNB

Biopsies were fixed with formaldehyde 4% during 24 h, and then paraffin-embedded. From each block, 7 cuts of 2.5 μm thick were performed. Histological preparations were stained using Hematoxylin and Eosine, Masson's trichrome (for assessing fibrosis and collagen deposition), and Weigert-Van-Giesson (for the architectural evaluation of elastic fibers). The slides were scanned using a 3D Histech P-1000 scanner, at 40x resolution, obtaining whole slide images. Histological diagnosis was performed by two experienced pulmonary pathologists (blinded to clinical and radiological findings).

### Statistical Analysis

Categorical data were described as frequency and percentages. Continuous variables were expressed as mean and standard deviation for normally distributed variables or median and interquartile range otherwise. Positive and negative predictive values (PPV and NPV) were calculated. Data were analyzed by using SPSS 24 (SPSS, IBM Corp) and R (Software Version 3.6.2).

## Results

Thirty consecutive patients admitted into the IMCU that presented disease worsening were included in the study. The median time between CT scan and LU was 5 days (IQR, 12.5 days) and between CT scan and histology was 8.4 days (IQR, 9 days). Histological samples were taken LU-guided. The mean age was 62.56 ± 13.27 years old, with 29 (96.7%) male patients. Patients received complete COVID-19 treatment, following the approved protocol. Thirteen patients died after 30 ± 16.8 days of inclusion. Those who died were older than those who recovered and presented a higher prevalence of smoking history and comorbidity. The most frequent comorbidities were hypertension (70%), dyslipidemia (53.3%), and history of malignancies (40%). The mean time from hospitalization to death was 30 ± 16.89 days. The main cause of death was respiratory failure (*n* = 11; 84.61%), multiple organ dysfunction (*n* = 1; 7.69%), and refractory septic shock (*n* = 1; 7.69%). The demographic and clinical characteristics of included patients are described in Table 1.

**Table 1:** Demographic and clinical characteristics of patients at the time of evaluation according to lung ultrasound score.

	**Total of patients (*n* = 30)**	**Progressive worsening and death (*n* = 13)**
**Patient background**		
Age, mean (SD)	62.56 (13.27)	67.92 (12.96)
Male, *n* (%)	29 (96.7%)	9 (69.23%)
Smoking history, *n* (%)	9 (30%)	5 (38.48%)
Hypertension, *n* (%)	21 (70%)	9 (69.23%)
Dyslipidemia, *n* (%)	16 (53.3%)	5 (38.48%)
Diabetes, *n* (%)	9 (22.50%)	4 (30.77%)
Obesity, *n* (%)	15 (30%)	4 (30.77%)
COPD, *n* (%)	3 (10%)	1 (7.69%)
Asthma, *n* (%)	2 (6.7%)	1 (7.69%)
Cardiopathy, *n* (%)	2 (6.7%)	1 (7.69%)
Hepatopathy, *n* (%)	2 (6.7%)	0
History of malignancies, *n* (%)	8 (40%)	5 (38.48%)
Chronic kidney disease, *n* (%)	3 (10%)	1 (7.69%)
**Laboratory blood test on LU initial assessment**		
Lactate dehydrogenase (U/L), median (IQR)	409 (205.50)	426 (136)
C-Reactive protein (mg/L), median (IQR)	66.90 (146.60)	159.5 (219.50)
Ferritin (μg/L), median (IQR)	985.90 (932.10)	1,237 (2,198.50)
Leukocyte count (x10^9^/L), median (IQR)	12.25 (10.75)	15.4 (15.60)
Lymphocyte count (x10^9^/L), median (IQR)	0.93 (0.89)	0.73 (0.68)
D-dimer (μg/L), median (IQR)	707 (641.50)	735 (1,480)

*LUS, lung ultrasound score; COPD, chronic obstructive pulmonary disease; SD, standard deviation; IQR, interquartile range*.

### LU and CT Scan Comparative

**Figure 1:**
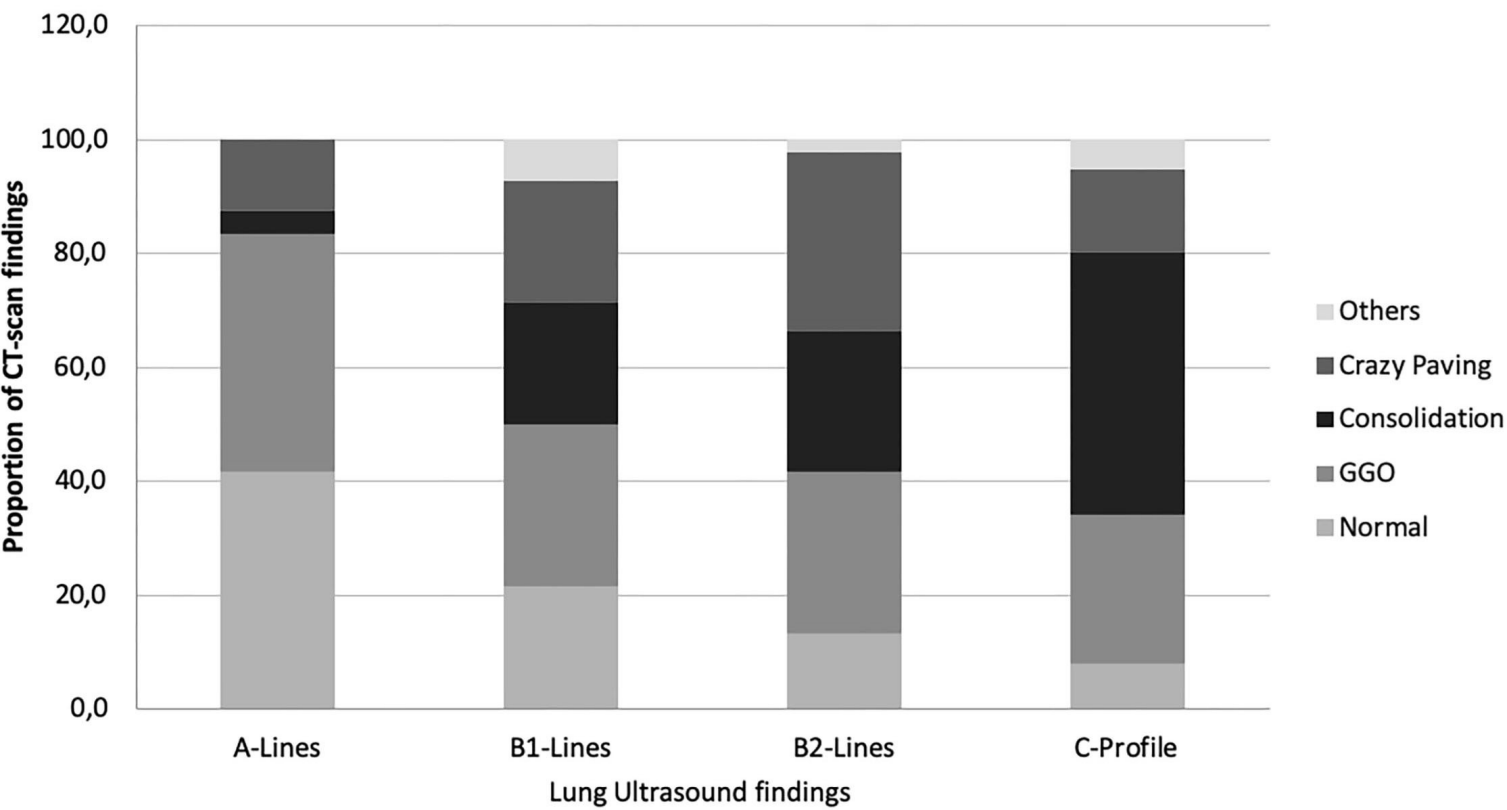
Proportion of CT-scan findings in the function of LU images. CT scan, computerized tomography scan; LU, lung ultrasound; GGO, ground-glass opacity.

Lung ultrasound and CT scan were both performed in 30 patients, with a total of 279 thoracic regions explored. In 4 (13.33%) patients, the complete twelve-zone ultrasonographic exploration was not possible. The most frequent LU finding was B2-lines (137/279 thoracic regions; 49.1%), followed by C-profile (76/279; 27.2%), B1-lines (42/279; 15.1%), and A-lines (24/279; 8.6%). Average LUS was 23.5 ± 6.2 points, which reveals a moderate–severe alveolar–interstitial pattern. The most frequent CT-scan finding was GGO (81/279 thoracic regions; 29%), followed by consolidation (79/279; 28.3%), and crazy paving (66/279; 23.7%). Interestingly, the type of LU finding in the different regions may predict CT-scan abnormalities; while A-lines were associated with areas of normal lung aeration in 10/24 of observation regions (41.7%), pathological CT-scan findings were commonly observed when B2-lines or C-lines were identified through LU (189/213; 88.7%). The type of radiological CT pattern was variable in those cases that presented C-profile: consolidation (35/76; 46.1%), GGO (20/76; 26.3%), and crazy paving (11/76; 14.5%); instead, some area of the normal lung was only observed in 6/76 (7%). LU and CT-scan findings are shown in Table 2 and Figure 1.

**Table 2:** Description of all thoracic regions explored by LU and CT scan.

					**CT-SCAN**			
		**Normal**	**GGO**	**C**	**CP**	**PT**	**PE**	**Total explored regions (%)**
LU	A-Lines *N* (%)	10 (41.7%)	10 (41.7%)	1 (4.2%)	3 (12.5%)	0	0	24 (8.6%)
	B1-Lines *N* (%)	9 (21.4%)	12 (28.6%)	9 (21.4%)	9 (21.4%)	1 (2.4%)	2 (4.8%)	42 (15.1%)
	B2-Lines *N* (%)	18 (13.1%)	39 (28.5%)	34 (24.8%)	43 (31.4%)	0	3 (2.2%)	137 (49.1%)
	C-Profile *N* (%)	6 (7.9%)	20 (26.3%)	35 (46.1%)	11 (14.5%)	0	4 (5.3%)	76 (27.2%)
	Total explored regions (%)	43 (15.4%)	81 (29.0%)	79 (28.3%)	66 (23.7%)	1 (0.4%)	9 (3.2%)	279 (100%)

*The proportion of CT-scan findings in relation to LU findings in the same explored regions. CT scan, computerized tomography scan; GGO, ground-glass opacity; C, consolidation; CP, crazy paving; PT, pneumothorax; PE, pleural effusion; LU, lung ultrasound*.

### Histological Findings

**Figure 2:**
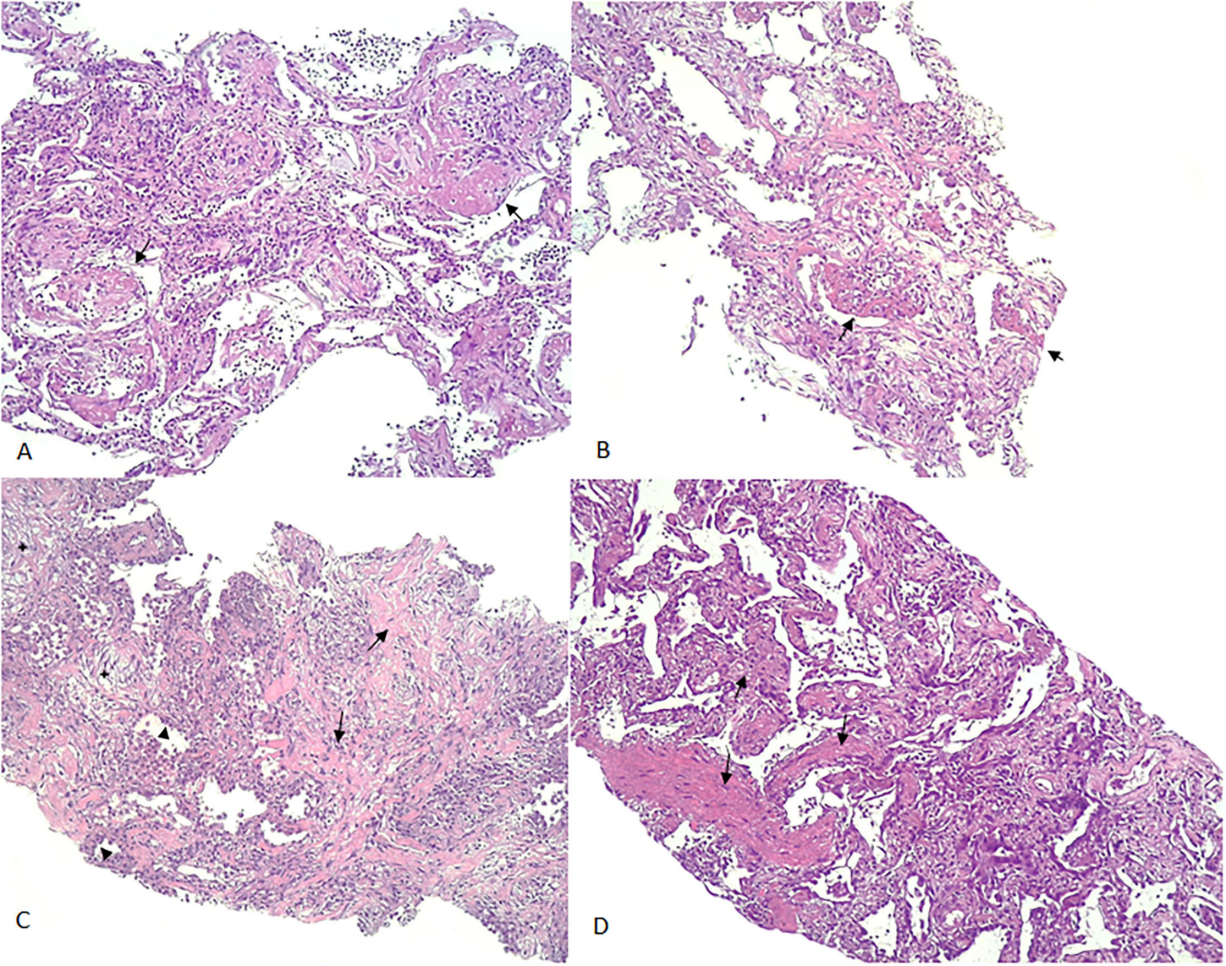
Examples of CNB. **(A)**: AFOP-like DAD: (arrows: deposition of fibrin plugs filling the alveolar space); **(B)**: AFOP-like organizing DAD (arrows: fibrin plugs deposited in the alveoli and focal organization changes in the interstitium); **(C)**: AFOP-like fibrotic DAD [focal organizational changes (star), interstitial deposition of collagen (arrow) accompanied by pneumocyte type II hyperplasia (arrowhead)]. **(D)**: Fibrotic DAD (arrows: thickening of the alveolar septa with mature collagen deposition in the interstitium). CNB, core needle biopsy; DAD, diffuse alveolar damage; AFOP, acute fibrinous organizing pneumonia.

Twenty-five echo-guided histological samples were obtained from 13 patients. All patients presented the same LU findings in both hemithoraxes. Samples obtained from one patient were not representative of lung parenchyma. From the remaining 24 samples, 18 (75%) showed DAD, whereas 6 (25%) showed chronic interstitial inflammation. The observed DAD was heterogeneous, showing multiple evolving patterns of damage, including exudative (8/24, 33.3%), fibrotic (8/24, 33.3%), and organizing (2/24, 8.3%) phases. In those patients with acute or exudative pattern, two lesions were distinguished: classic hyaline membrane and fibrin “plug” in alveolar space (acute fibrinous organizing pneumonia, AFOP). Regarding fibrotic findings, collagen fibrosis was predominant, frequently associated to pneumocyte hyperplasia. Consistent fibrosis with architectural remodeling was seen in only two samples (8.3%). Neither microangiopathy nor microthrombi was found. Examples of CNB are shown in Figure 2.

### Lung Ultrasound and Histological Findings

**Figure 3:**
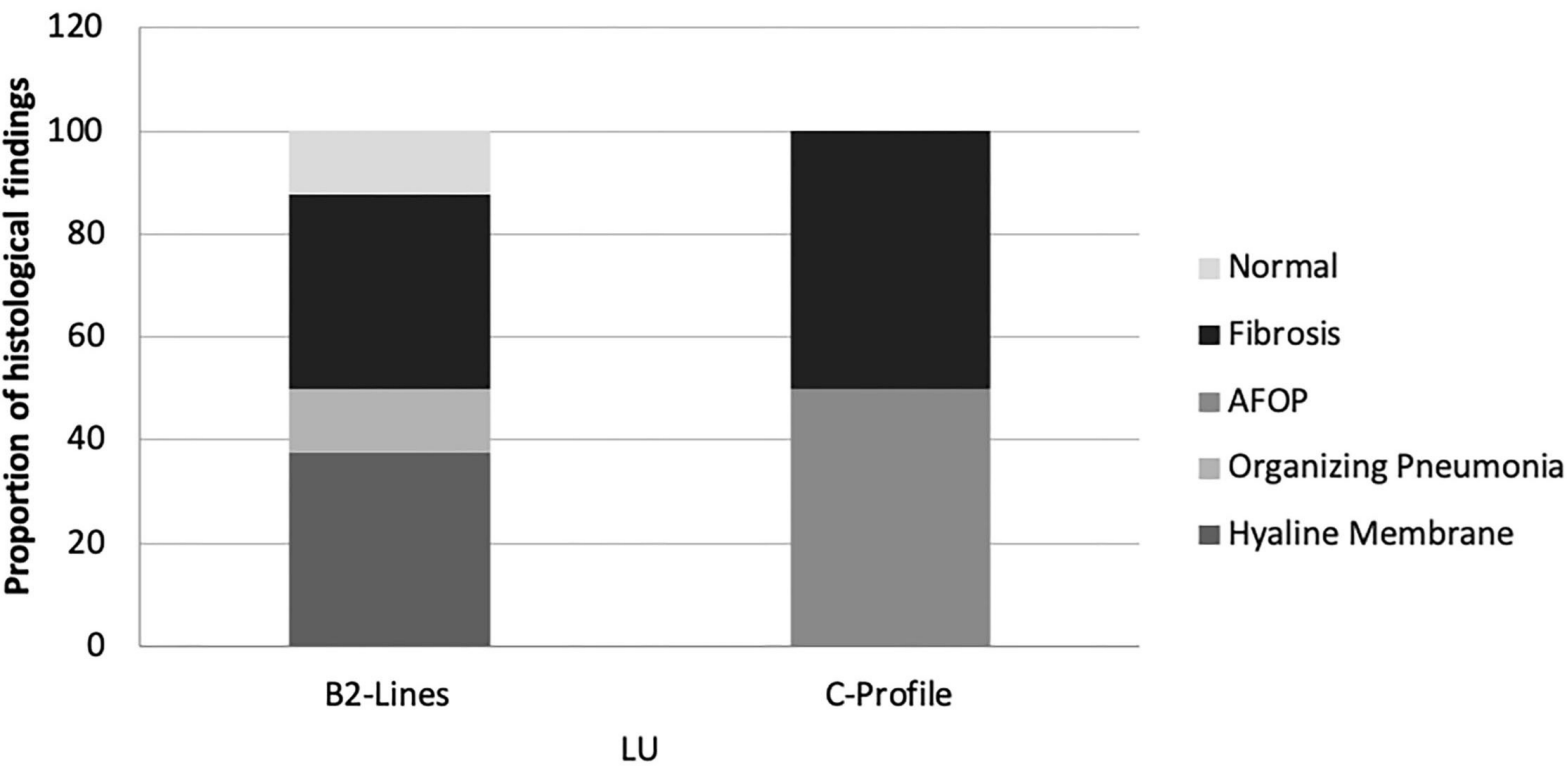
Proportion of histological findings in function of LU images. AFOP, Acute Fibrinous Organizing Pneumonia; LU, Lung ultrasound.

Lung ultrasound and histological findings were matched in the 12 patients with representative lung biopsy samples. In these cases, only C-profile and B2-lines were identified. C-profile was described in 4 patients (33.3%). These cases presented histological signs of DAD and lung fibrosis. The predominant finding in 2 (50%) of them was collagen deposition and AFOP in the other two cases. B2-lines were identified in 16 regions (66.7%); the presence of hyaline membrane was the predominant finding in 6 of them (37.5%), organizing pneumonia in 2 samples (12.5%), and fibrosis in 6 samples (37.5%). No A-lines or B1-lines were observed in these patients (see Figure 3).

## Discussion

The present results show that a majority of patients with severe COVID-19 with worsening of respiratory failure present B2-lines and C-lines in LU, which histologically correspond to different signs of DAD, including hyaline membranes and AFOP areas, and lung fibrosis. GGOs and crazy-paving areas in the CT had no specific LU imaging correlation since both were found in all LU patterns. However, CT areas of consolidation were more predominant in LU B2-lines and C-lines. Those patients who died because of respiratory failure only presented LU B2-lines and C-lines at the time of death.

In 2008, an LU protocol was introduced in the examination of acute respiratory failure: the BLUE (Bedside Lung Ultrasound in Emergency) protocol (25, 26). The accuracy of this protocol in diagnosing the cause of acute respiratory failure in critical care patients was 90.5%, with higher sensitivity, specificity, and diagnostic accuracy for pleural effusions, alveolar consolidation, and interstitial syndromes (12). Using the BLUE protocol features, COVID-19 causes clear and typical ultrasonographic patterns. B-lines in COVID-19 are predominant; both in separate (B1) and coalescent (B2) forms, and can give the appearance of a shining white lung. Irregularity of the pleural line and subpleural consolidations (C-profile) also occur in bilateral patchy clusters, and are mainly visible in the posterior and inferior areas (24, 26–28). According to published data, our results show that B2-lines were predominant in patients with severe COVID-19 followed by C-profile. These findings were able to predict the clinical outcome in patients admitted into the IMCU (28): Furthermore, the present data agree with previous findings which indicated that the higher the LUS, the worse clinical outcome is expected, but it also correlates with more pathological CT findings. Therefore, LUS could help in monitoring the patient clinical evolution in a bedside setting and help in the CT-scan indication.

Yang et al. (29) performed a two-centered retrospective comparison between LU and CT-scan imaging in hospitalized patients with COVID-19. In total, 63% of positive regions were detected by LU. In contrast, CT scan showed 38.7% of regions with abnormalities. Both LU and CT-scan findings showed the lesions of COVID-19 were more likely to occur in the posterior regions of the lung with bilateral distribution. In our study, LU detected alveolar–interstitial disorders in 91% (255/279), whereas CT scan detected in 84% (236/279). Our study shows higher radiological abnormalities because only patients with severe COVID-19 were included. When A-lines were described in LU, pathological CT-scan findings were observed in 58.3% of regions. Therefore, the finding of A-lines in patients with COVID-19 should not be interpreted as normal aeration of the lung but the probability of pathological CT-scan findings is lower than that for B-lines or C-profile. A-lines had an NPV of 87.1%. However, it has a PPV of 41.7% for normal lung aeration. Though, when C-profile is seen in LU, it is mainly associated with consolidation in CT-scan, but also with GGO and crazy paving. However, it is almost never associated with normal healthy lung (PPV 76% and NPV 18.3% for abnormal CT scan).

**Figure 4:**
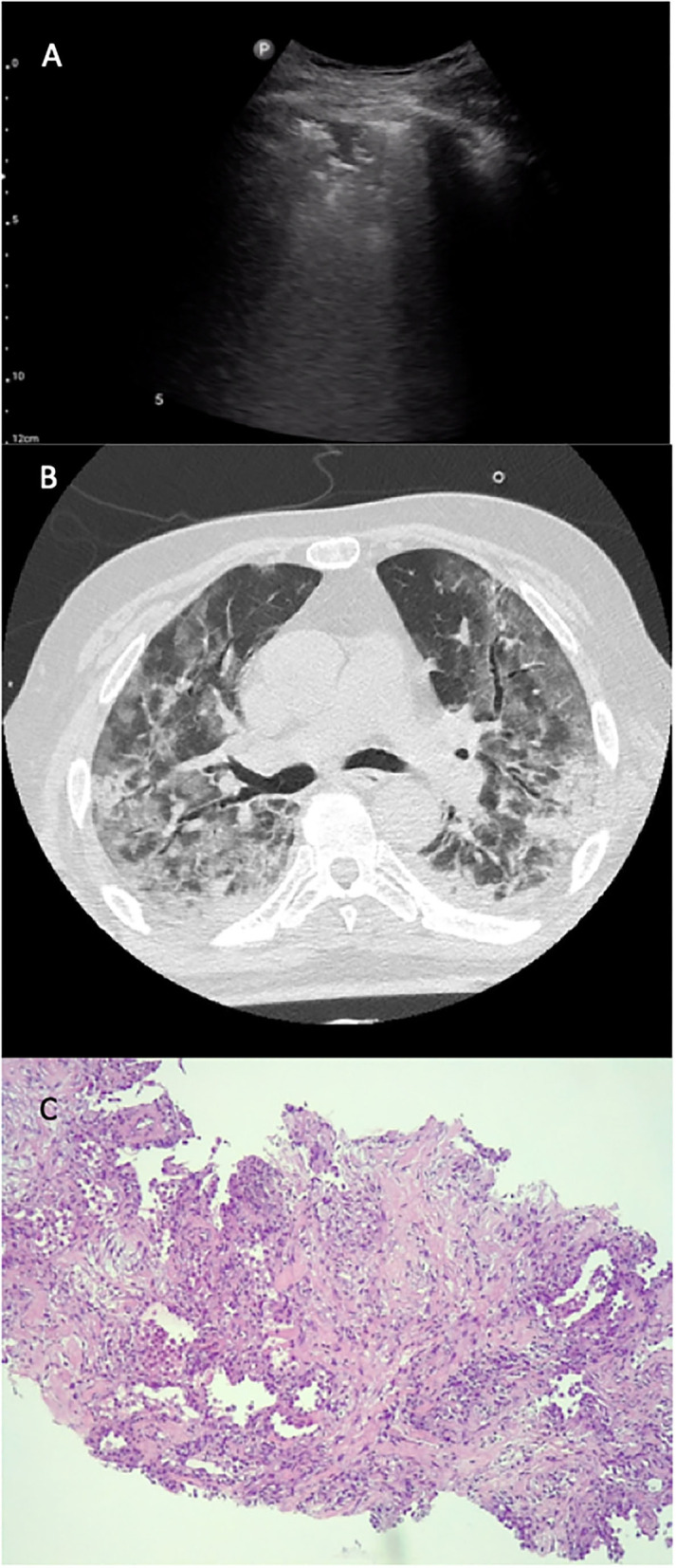
LU–CT–histology from the same region. LU **(A)**, CT-scan **(B)**, and histological **(C)** images of the same patient. The LU exploration **(A)** shows a C-profile pattern, seen in the anterior left region of the thorax; CT scan **(B)** shows areas of ground-glass opacity in the same region. In the histological sample **(C)**, we observe an AFOP-like fibrotic DAD (focal organizational changes, interstitial deposition of collagen accompanied by pneumocyte type II hyperplasia).

The initial recommendations for biosafety control required during specimen collection and handling have strongly limited the practice of autopsies of the COVID-19 at the beginning of this pandemic. The first autopsy study published by a Chinese group in February 2020 reported a case of postmortem CNB (18). Since then, the number of autopsies has increased. The main reported pathological findings were exudative DAD, as well as organizing pneumonia (OP), reactive type II pneumocytes, and chronic interstitial pneumonia (30, 31). The results of our CNB specimens are consistent with published data: the main histological finding was DAD (75%), including different pathological findings, such as AFOP areas together with collagen fibrotic areas and chronic interstitial inflammation (25%) (see Figure 4).

To date, there are no published data concerning the association between LU and histological findings in COVID-19. In our study, the LU exploration of only progressive severe COVID-19 cases showed B2-lines and C-profile. Recently, a high LUS (predominant B2-lines and C-profile) has been related to poor clinical outcomes (28). The present data show that the histological findings associated with C-profile are predominantly AFOP and collagen fibrosis, both findings associated with a poor prognosis and lack of response to glucocorticoid or immunomodulators (32).

The main limitation of our study is the reduced number of patients due to the difficulties in achieving both LU and CT images at the proper timeline of respiratory worsening in patients with COVID-19 and the challenges of the immediately postmortem LU-guided CNBs.

## Conclusion

LU B2-lines and C-profile are predominantly identified in patients with severe COVID-19 with respiratory worsening, which correspond to different CT patterns and histological findings of DAD and lung fibrosis. LU could help in optimizing CT requirements and anticipating prognosis or treatment response.

## Data Availability Statement

The raw data supporting the conclusions of this article will be made available by the authors, without undue reservation.

## Ethics Statement

The studies involving human participants were reviewed and approved by Comité de Ética de la Investigación del Hospital Universitari de Bellvitge (Re. N° PR122/20). Written informed consent for participation was not required for this study in accordance with the national legislation and the institutional requirements.

## Author Contributions

PT-S, ED, MM-M, and GS-C conceptualized the study and investigated the study. PT-S, ED, and GS-C contributed to data curation. GS-C contributed to formal analysis. PT-S, ED, MM-M, JD, SS, and GS-C contributed to methodology. PT-S, MM-M, and GS-C contributed to project administration. ED, SA, MD-F, JB-M, AM, JS, BR, XS, and PL contributed to resources. PT-S wrote the original draft. ED, MM-M, and GS-C contributed to writing, reviewing, and editing the manuscript. All authors contributed to the article and approved the submitted version.

## Funding

We thank CERCA programme for the financial support.

## Conflict of Interest

The authors declare that the research was conducted in the absence of any commercial or financial relationships that could be construed as a potential conflict of interest.

## Publisher's Note

All claims expressed in this article are solely those of the authors and do not necessarily represent those of their affiliated organizations, or those of the publisher, the editors and the reviewers. Any product that may be evaluated in this article, or claim that may be made by its manufacturer, is not guaranteed or endorsed by the publisher.
